# PRAME expression and promoter hypomethylation in epithelial ovarian cancer

**DOI:** 10.18632/oncotarget.9977

**Published:** 2016-06-13

**Authors:** Wa Zhang, Carter J. Barger, Kevin H. Eng, David Klinkebiel, Petra A. Link, Angela Omilian, Wiam Bshara, Kunle Odunsi, Adam R. Karpf

**Affiliations:** ^1^ Eppley Institute for Cancer Research, University of Nebraska Medical Center, Omaha, NE, USA; ^2^ Department of Biochemistry and Molecular Biology, University of Nebraska Medical Center, Omaha, NE, USA; ^3^ Department of Pharmacology, Roswell Park Cancer Institute, Buffalo, NY, USA; ^4^ Department of Biostatistics and Bioinformatics, Roswell Park Cancer Institute, Buffalo, NY, USA; ^5^ Department of Pathology, Roswell Park Cancer Institute, Buffalo, NY, USA; ^6^ Department of Gynecologic Oncology, Roswell Park Cancer Institute, Buffalo, NY, USA; ^7^ Department of Immunology, Roswell Park Cancer Institute, Buffalo, NY, USA; ^8^ Center for Immunotherapy, Roswell Park Cancer Institute, Buffalo, NY, USA; ^9^ Fred and Pamela Buffett Cancer Center, University of Nebraska Medical Center, Omaha, NE, USA; ^10^ Current address: Wilmer Eye Institute, Johns Hopkins University School of Medicine, Baltimore, MD, USA

**Keywords:** PRAME, cancer testis antigens, epithelial ovarian cancer, high grade serous cancer, DNA methylation

## Abstract

PRAME is a cancer-testis antigen (CTA) and potential immuno-therapeutic target, but has not been well-studied in epithelial ovarian cancer (EOC) or its high grade serous (HGSC) subtype. Compared to normal ovary, *PRAME* expression was significantly increased most EOC, regardless of stage and grade. Interestingly, *PRAME* mRNA expression was associated with improved survival in the HGSC subtype. The *PRAME* locus was a frequent target for copy number alterations (CNA) in HGSC but most changes were heterozygous losses, indicating that elevated *PRAME* expression is not typically due to CNA. In contrast, *PRAME* promoter DNA hypomethylation was very common in EOC and HGSC and correlated with increased *PRAME* expression. *PRAME* expression and promoter hypomethylation both correlated with LINE-1 hypomethylation, a biomarker of global DNA hypomethylation. Pharmacologic or genetic disruption of DNA methyltransferase (DNMT) enzymes activated *PRAME* expression in EOC cells. Immunohistochemistry (IHC) of PRAME in EOC revealed frequent, but low level, protein expression, and expression was confined to epithelial cells and localized to the cytoplasm. Cytoplasmic PRAME expression was positively associated with *PRAME* mRNA expression and negatively associated with promoter methylation, but the latter correlation was not statistically significant. PRAME protein expression did not correlate with EOC clinicopathology or survival. In summary, *PRAME* is frequently expressed in EOC at the mRNA and protein levels, and DNA methylation is a key mechanism regulating its expression. These data support PRAME as an immunotherapy target in EOC, and suggest treatment with DNMT inhibitors as a means to augment PRAME immunotherapy.

## INTRODUCTION

Ovarian cancer is the seventh most common female cancer worldwide and accounts for the fifth most female cancer deaths in the US [1, 2]. Approximately 90% of ovarian cancer cases are epithelial (EOC) [3]. More than 60% of EOC are diagnosed with advanced disease, and the five-year survival for these women is < 30% [2]. High grade serous cancer (HGSC) is the most common EOC subtype, accounting for ~70% of cases. HGSC is often clinically-advanced (stage 3+), is high grade (grade 2+), and is associated with poor survival [4]. Recent large-scale genomic studies have shed light on the molecular mechanisms of HGSC [4, 5]. Continued investigations are needed to facilitate improved diagnostic and therapeutic approaches.

*PRAME* (preferentially expressed antigen in melanoma; a.k.a. *MAPE*, *OIP4, CT130*) was identified as a gene encoding an HLA-A24-restricted peptide that stimulated tumor-specific cytotoxic T lymphocytes in a human melanoma cell line [6]. *PRAME* is located on chromosome 22q11.22 and encodes a 509 amino acid protein [7]. *PRAME* is an autosomal cancer-testis antigen (CTA) gene, based on its chromosomal location, expression profile, and immunogenicity. PRAME is not commonly expressed in normal adult somatic tissues, with the exception of testis [6], but is expressed in many cancers, and is immunogenic [8–10]. PRAME is expressed in both solid tumors and leukemia, making it an attractive potential immunotherapy target [11]. Similar to other CTAs *PRAME* has been reported to be epigenetically regulated by DNA methylation [12, 13]. Promoter hypomethylation of *PRAME* was reported in AML and MDS [14, 15]. In addition, the DNA methyltransferase inhibitor (DNMTi) 5-aza-2′-deoxycytidine (decitabine) induced *PRAME* expression in cancer cell lines [16–20]. Importantly, decitabine can also stimulate *PRAME*-specific immune-reactivity, suggesting an approach to augment PRAME immunotherapy, similar to our strategy for the X-linked CTA NY-ESO-1 [20–23].

In addition to its expression in cancer and immunogenicity, PRAME is of interest based on a possible contribution to oncogenesis. PRAME was reported to be a ligand-dependent RAR corepressor and inhibitor of retinoic acid (RA) signaling [24]. PRAME expressing leukemia cells were growth inhibited in a dose-dependent fashion by all trans retinoic acid (ATRA), suggesting a therapeutic approach for tumors that express PRAME [8, 25]. More recent studies have additionally linked PRAME to chemotherapy sensitivity and apoptosis [26–28]. PRAME expression has also been reported to be associated with prognosis, although this has been an inconsistent observation [27, 29–32].

In EOC, *PRAME* has not been well-studied, but initial reports indicate that it is expressed and may be associated with survival [32–35]. PRAME mRNA and protein expression in EOC were reported to correlate [32, 35], suggesting the importance of transcriptional regulation for PRAME expression in EOC. No information has been provided regarding the genetic or epigenetic mechanisms that account for PRAME expression in EOC. Also, the relationship between PRAME and the status of the RA pathway in EOC is unknown. Here we address these and other important gaps in our knowledge regarding PRAME in EOC and HGSC.

## RESULTS

### *PRAME* mRNA expression in EOC

**Figure 1:**
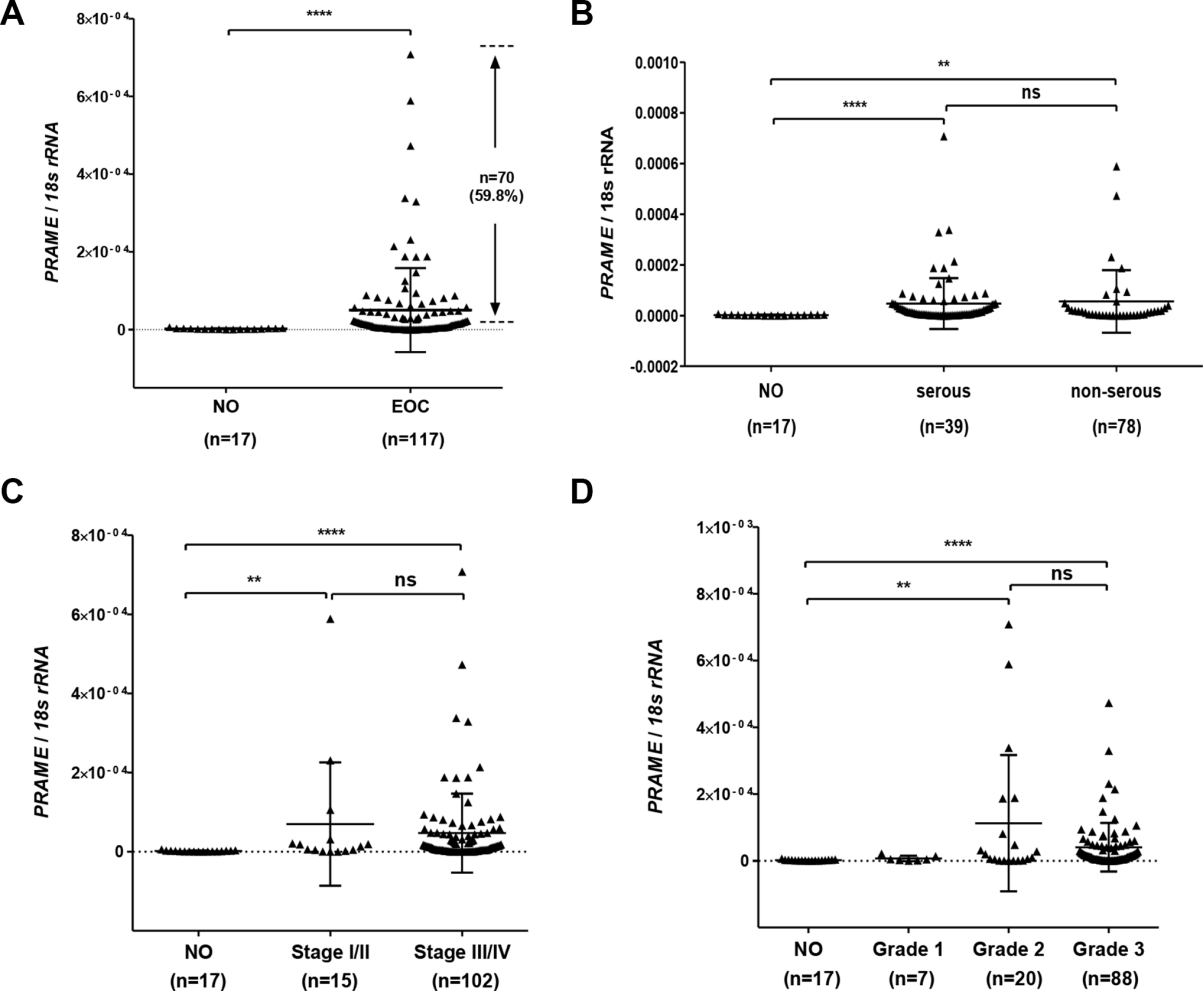
*PRAME* mRNA expression in EOC *PRAME* mRNA expression was measured by RT-qPCR and normalized to *18s rRNA*. (**A**) *PRAME* in normal ovary (NO) and EOC. The proportion of EOC with elevated *PRAME* mRNA expression as compared to NO is indicated. (**B**) *PRAME* in NO, serous histology EOC, and other histology EOC. (**C**) *PRAME* expression and EOC clinical stage, separated into Stage I/II and Stage III/IV. (**D**) *PRAME* expression and EOC histological grade. The two-tailed Mann-Whitney test *p*-value is indicated (**P < 0.01; ****P < 0.0001; ns: not significant).

We measured *PRAME* expression using RT-qPCR in a set of primary EOC (*n* = 119) and normal ovary (NO; *n* = 17) samples collected at Roswell Park Cancer Institute (RPCI; see *Methods*). *PRAME* was overexpressed in a majority of (~60%) of primary EOC as compared to NO (Figure 1A). *PRAME* mRNA was significantly increased expression both in serous and non-serous histology EOC (Figure 1B). *PRAME* was also significantly increased in both early (I/II) and advanced (III/IV) stage EOC, and in both grade 2 and grade 3 EOC (Figure 1C–1D).

### *PRAME* mRNA expression and HGSC survival

**Figure 2:**
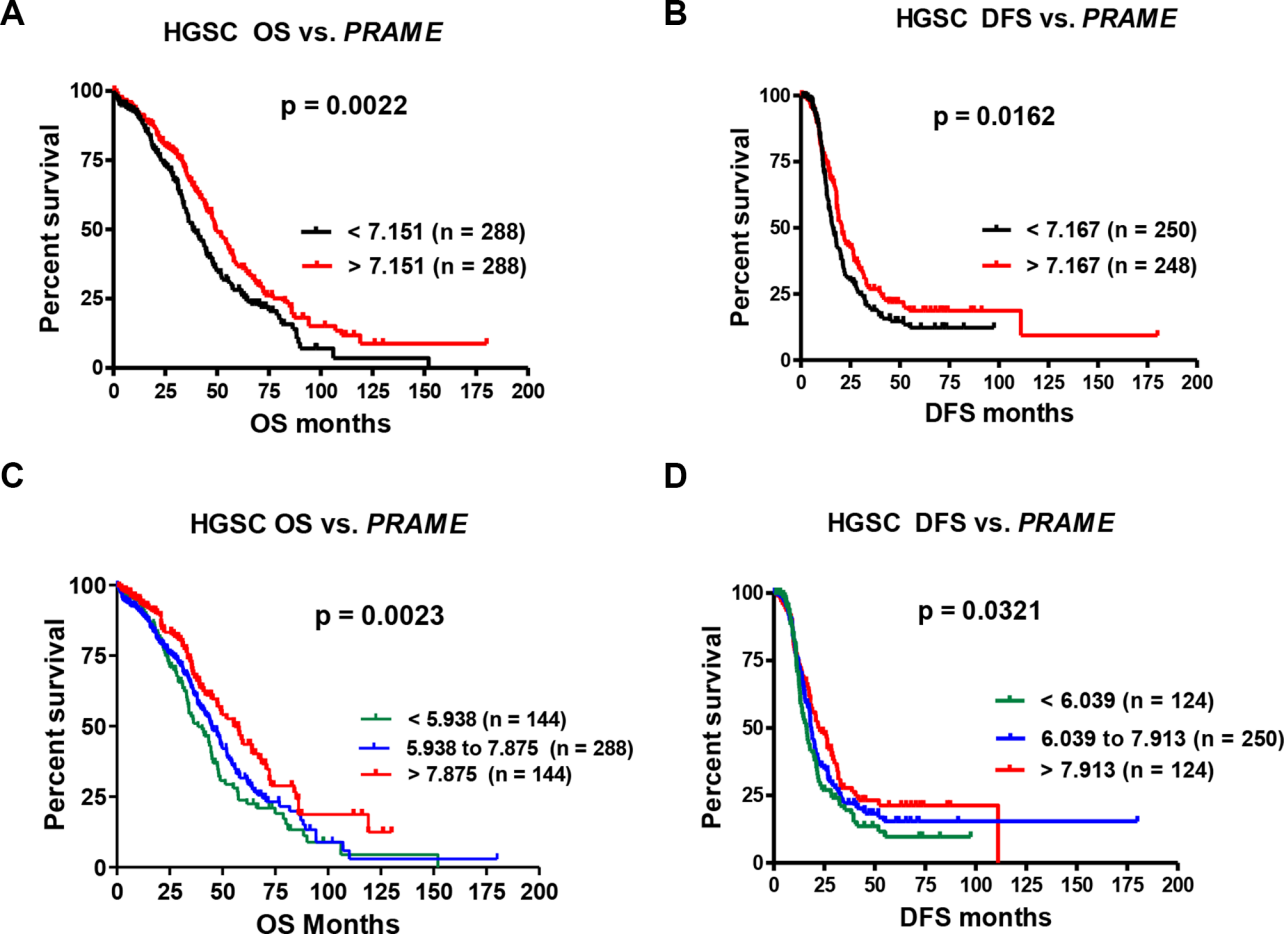
*PRAME* mRNA expression and HGSC patient survival (**A**) Overall survival (OS) and (**B**) Disease-free survival (DFS) of HGSC patients vs. *PRAME* expression, dichotomized at the median expression value. (**C**) OS and (**D**) DFS of HGSC patients vs. *PRAME* expression tertiles. *PRAME* expression was measured by Affymetrix U133 microarray; data were obtained for TCGA HGSC from cBioPortal. Logrank test *p*-values for two group comparison (A–B) or three-group trends (C–D) are shown.

Analysis of our EOC data did not reveal a significant association of *PRAME* mRNA expression with patient survival (data not shown). However, we had a limited number of patients evaluable for survival, and our samples were heterogeneous with regards to EOC histological subtype, which complicates survival analysis. Thus, we used data from The Cancer Genome Atlas (TCGA) [4]. TCGA includes only HGSC, and contains a large number of patients evaluable for survival. Restricting survival analysis to HGSC also largely mitigates the influence of disease progression, as the vast majority HGSC cases are clinically-advanced. We used three sources of mRNA expression data from TCGA, Affymetrix microarray (*N* = 576), Agilent microarray (*N* = 561), and RNAseq (*N* = 307), which we independently tested for survival associations. Using Affymetrix data, *PRAME* expression correlated with improved overall and disease-free (a.k.a. progression-free) survival (OS; DFS) (Figure 2). Similar results were obtained using Agilent data (OS, *p* = 0.036; DFS, *p* = 0.118). RNAseq data displayed a trend toward improved survival with increased *PRAME*, but this did not reach statistical significance (OS, *p* = 0.196; DFS, *p* = 0.694). The reason for the distinct results between microarray and RNAseq is unknown, but could relate to the number of samples analyzed.

### *PRAME* genomic copy number alterations in HGSC

**Figure 3:**
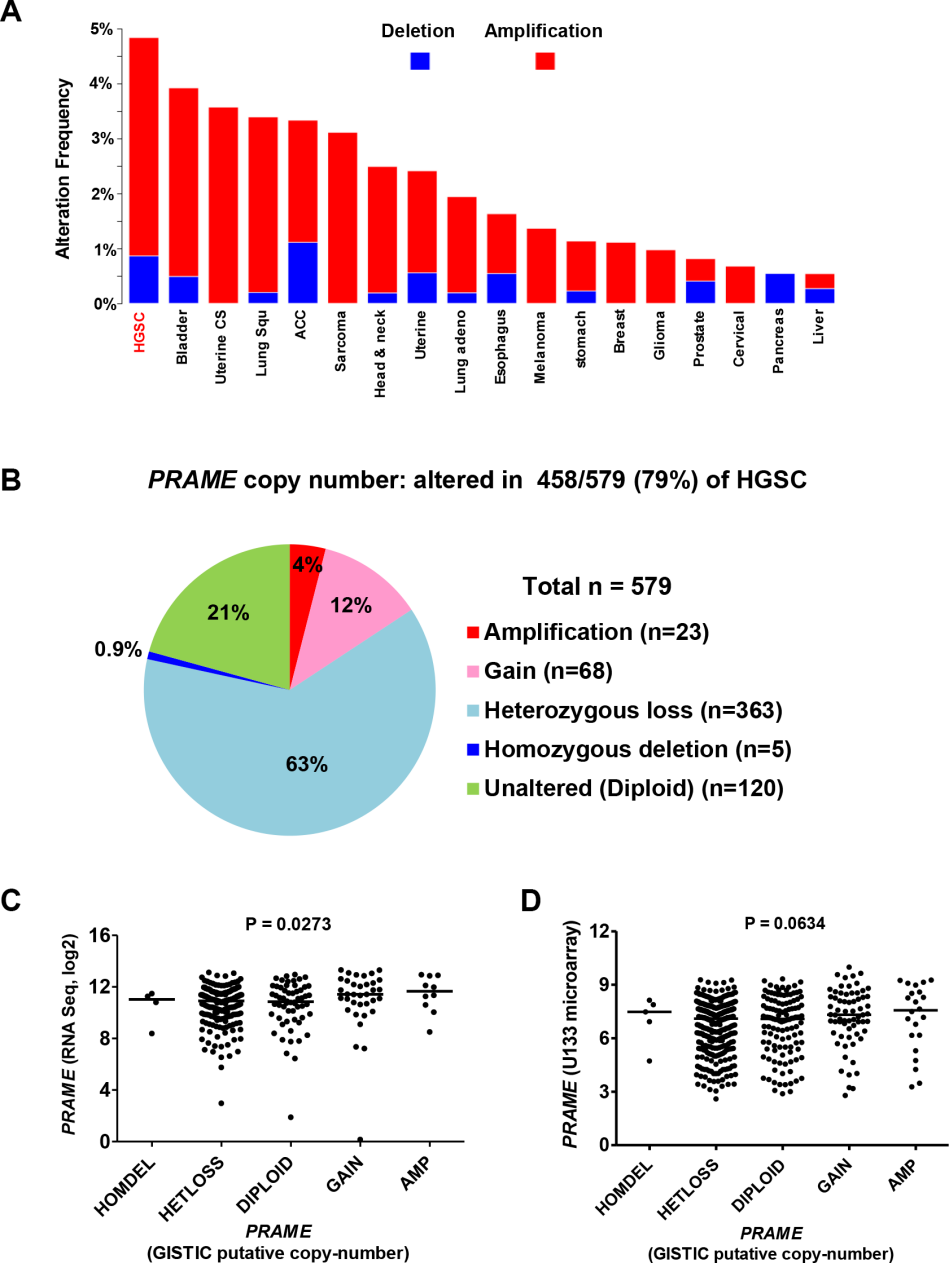
*PRAME* copy number and mRNA expression in HGSC TCGA data were obtained from cBioPortal [36]. (**A**) *PRAME* CNA (deletions-blue; amplifications-red) in different TCGA tumor types. The *PRAME* locus is at 22q11.22. (**B**) *PRAME* copy number data for HGSC (*n* = 579). (**C**) *PRAME* mRNA expression (RNAseq) and *PRAME* copy number in HGSC (*N* = 300). (**D**) *PRAME* mRNA expression (Affymetrix U133 microarray) and *PRAME* copy number in HGSC (*N* = 556). In C-D, Kruskal-Wallace *p*-values are shown.

To define the mechanisms underlying increased *PRAME* expression, we examined copy number alterations (CNA) using cBioPortal analysis of TCGA data [36, 37]. CNA are the predominant molecular alteration in HGSC and are associated with altered gene expression [4, 38, 39]. The chromosomal location of *PRAME* is 22q11.22. Amongst all tumor types with TCGA data, *PRAME* was most frequently amplified in HGSC (Figure 3A). However, the proportion of HGSC with *PRAME* amplification was relatively low (< 4.0%) as compared to the high prevalence of increased *PRAME* expression (Figure 3A; Figure 1). Interestingly, most HGSC cases show *PRAME* CNA, but the majority of these are heterozygous losses (63%), while copy number gains + amplifications are far less frequent (16%) (Figure 3B). *PRAME* expression significantly correlated with genomic copy number using RNAseq data, and showed borderline significance using microarray data (Figure 3C–3D). Based on the relationship between *PRAME* mRNA expression and survival as described above, we tested whether *PRAME* copy number correlated with HGSC survival. However, *PRAME* copy number (gains + amplifications vs. all other copy number states) did not correlate with HGSC survival (data not shown). Together, these data indicate that copy number influences *PRAME* expression in HGSC, but suggests that additional mechanisms are more likely to account for the increased expression of *PRAME* commonly observed in this disease.

### *PRAME* promoter region DNA methylation in EOC

**Figure 4:**
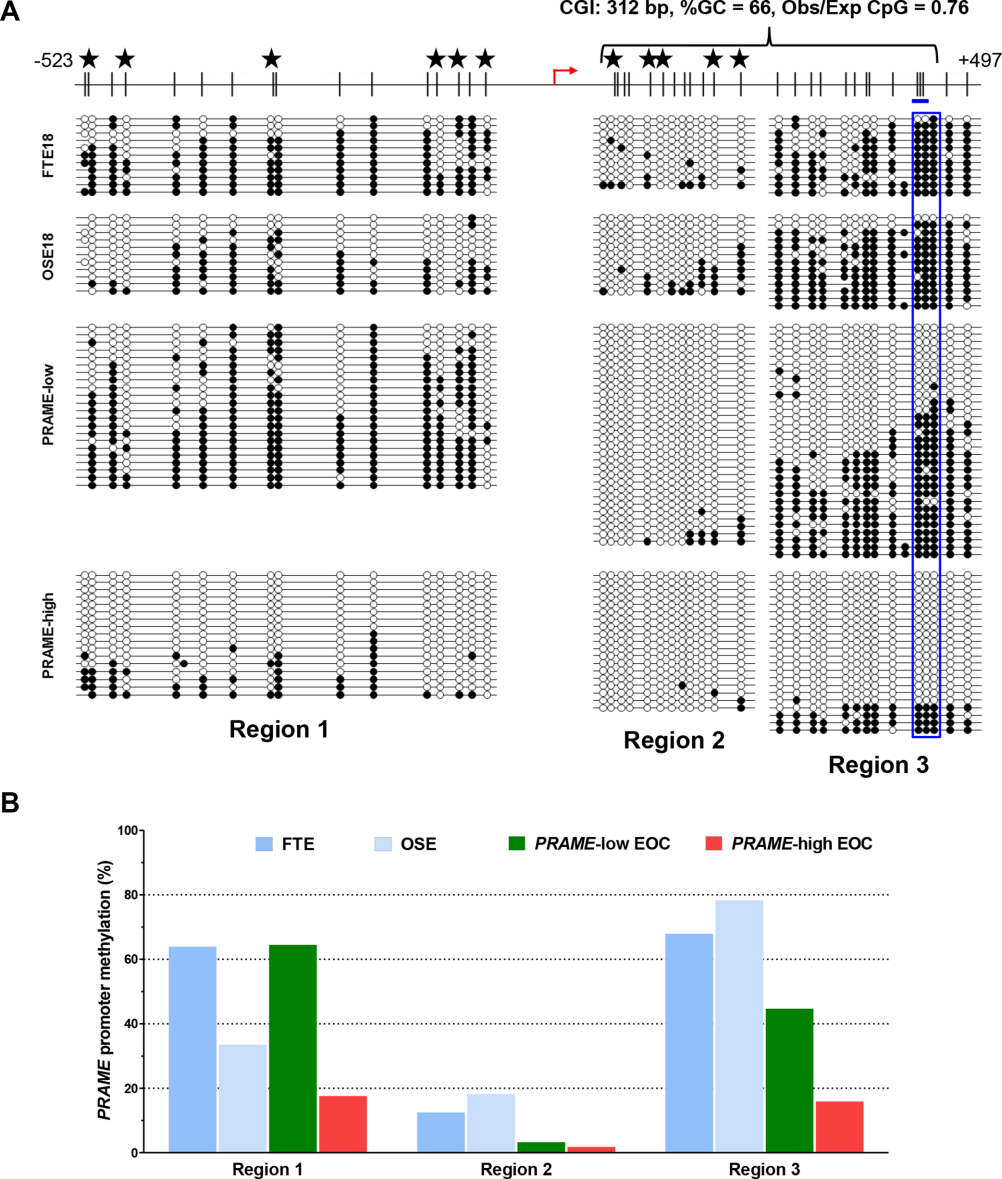
Sodium bisulfite clonal sequencing of the *PRAME* 5′ region (**A**) Top: diagram of the *PRAME* promoter and 5′UTR region, including CpG sites (black hash marks), NCBI-predicted TSS (red broken arrow), and region analyzed by pyrosequencing (blue rectangle). Stars indicate overlapping CpG analyzed by Illumina 450 K bead arrays (see Figure 5C). Bottom: bisulfite clonal sequencing data for three consecutive regions of the *PRAME* 5′ region. Filled and open circles indicate methylated and unmethylated CpGs, and each row represents one sequenced allele. Data are shown for FTE, OSE, a pool of three EOC samples with low *PRAME* expression, and a pool of three EOCs with high *PRAME* expression. (**B**) Graphical summary of data presented in (A).

Promoter DNA methylation has been shown to regulate the expression of both X-linked and autosomal CTA genes in EOC [13, 40–42]. To examine whether *PRAME* is regulated by DNA methylation in EOC, we first examined the *PRAME* gene structure and observed that the 5′ region of *PRAME*, which overlaps the promoter and 5′ untranslated region (UTR), contains a CpG island [43] located downstream of the transcriptional start site (TSS) (Figure 4A, top). To determine if promoter methylation correlates with *PRAME* expression in EOC, we conducted bisulfite clonal sequencing to survey three areas of the 5′ region of *PRAME* in normal controls and EOC (Figure 4A). We analyzed methylation of four sample groups: 1) normal fallopian tube epithelium (FTE), 2) normal ovarian surface epithelium (OSE), 3) EOC sample pool (*n* = 3) with low *PRAME* expression and 4) EOC sample pool (*n* = 3) with high *PRAME* expression. Notably, *PRAME* methylation was significantly reduced throughout the 5′ region in EOC tumors expressing high levels of *PRAME*, as compared to the normal controls or to EOC showing low *PRAME* expression (Figure 4A–4B).

**Figure 5:**
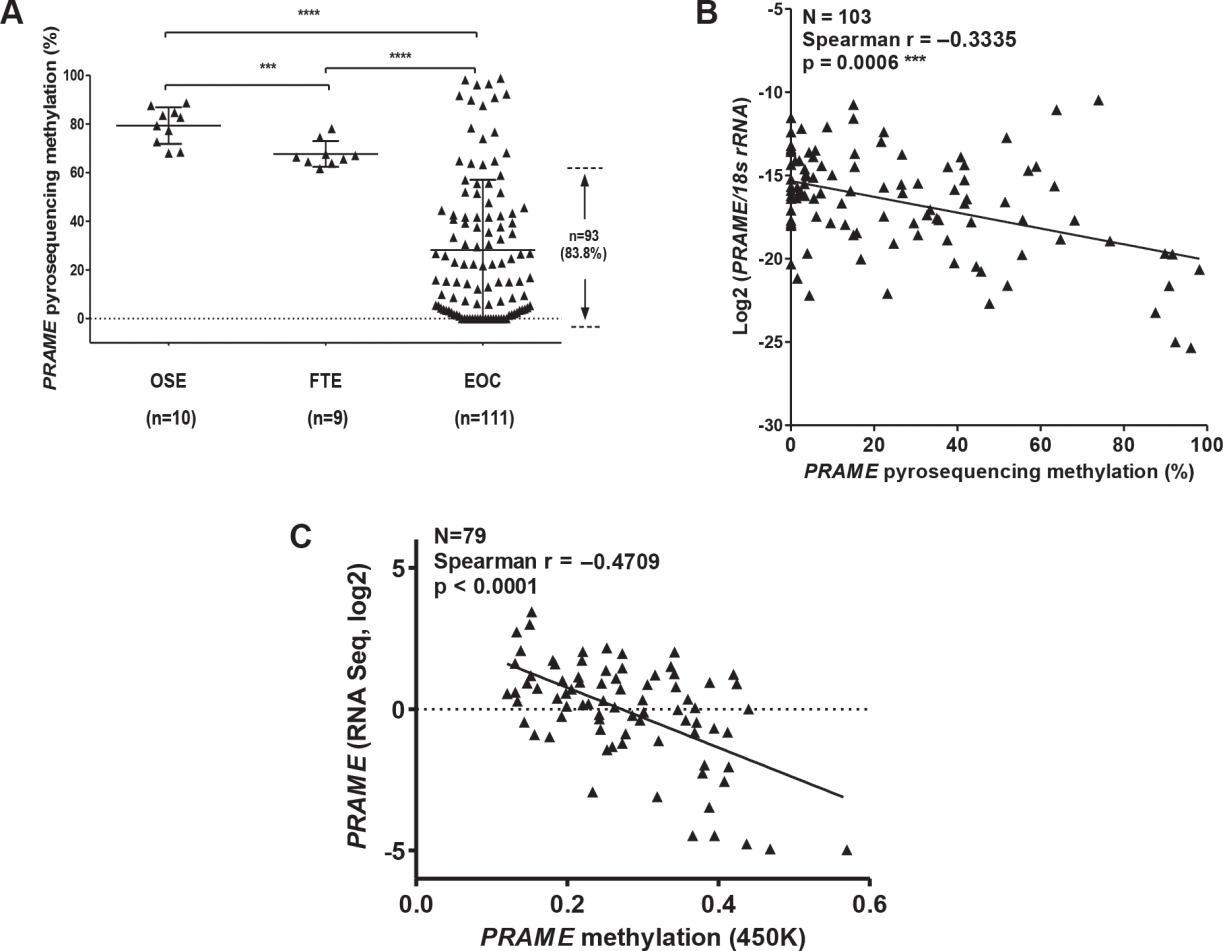
Sodium bisulfite pyrosequencing of *PRAME* 5′ region and *PRAME* mRNA expression (**A**) Pyrosequencing methylation data for three CpGs (averaged) (see blue rectangle in Figure 4A) in OSE, FTE, and EOC. The proportion of EOC with *PRAME* methylation lower than all OSE and FTE samples is indicated. The two-tailed Mann-Whitney test *p*-value is indicated (***P < 0.001; ****P < 0.0001). (**B**) Association of *PRAME* methylation and expression. *PRAME* mRNA expression was measured by RT-qPCR and was normalized to *18s rRNA* expression. *PRAME* promoter methylation was measured by pyrosequencing. Spearman test results (two-tailed) are shown. (**C**) *PRAME* methylation and mRNA expression in ICGC data. Methylation was determined in primary HGSC tumors using Illumina 450 K arrays and *PRAME* mRNA expression was determined using RNAseq. The CpG sites measured by this assay are indicated by stars in Figure 4A. Spearman test results (two-tailed) are shown.

**Figure 6:**
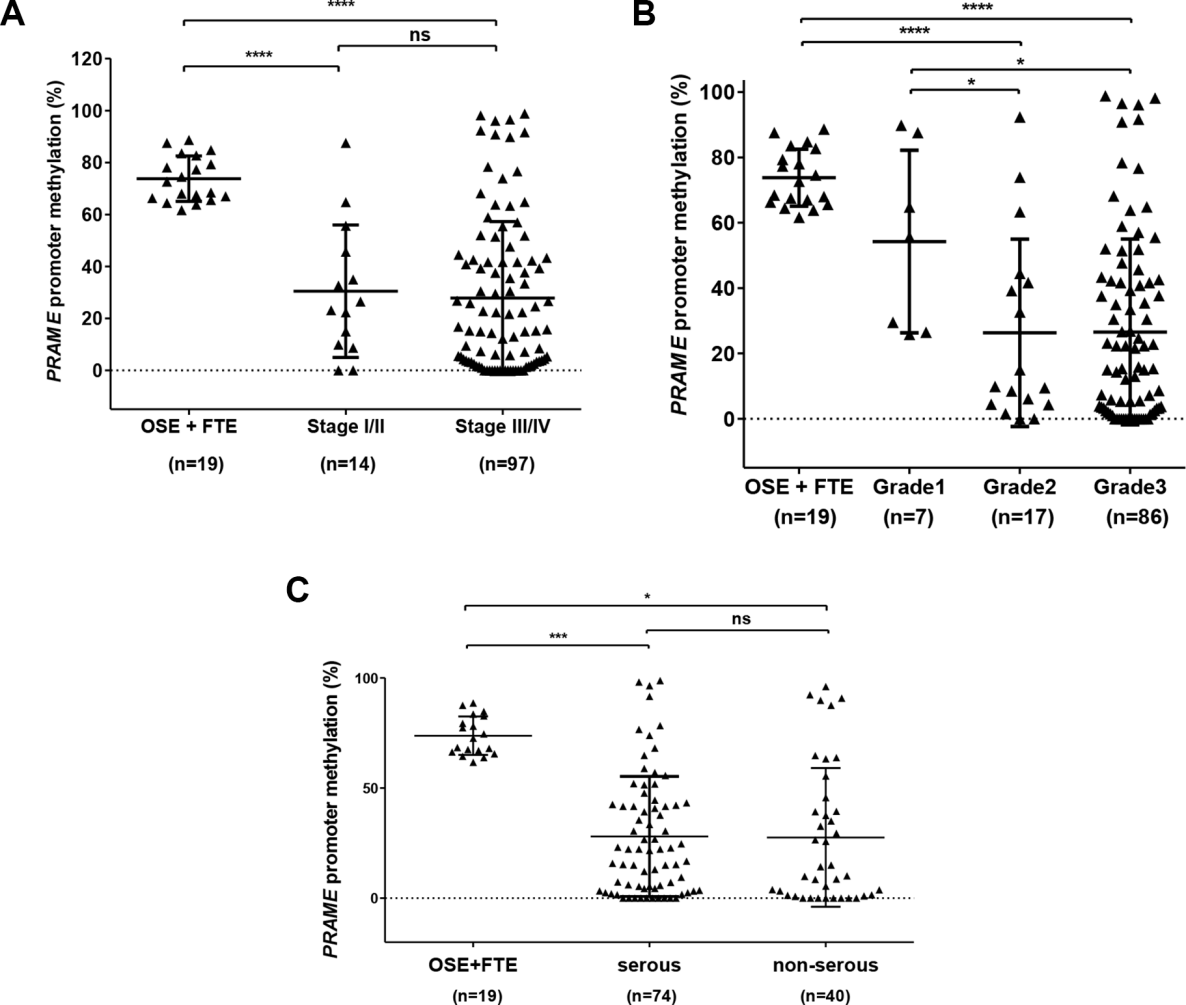
*PRAME* methylation and EOC stage, grade, and histology (**A**–**B**) *PRAME* methylation was measured by pyrosequencing in OSE, FTE, and EOC, and data are shown as a function of EOC stage (A) and grade (B). (**C**) *PRAME* methylation in OSE + FTE, serous EOC, and non-serous EOC. The two-tailed Mann-Whitney test *p*-value is indicated (*P < 0.05; ***P < 0.001; ****P < 0.0001; ns: not significant).

Bisulfite clonal sequencing is a low-throughput assay poorly suited for interrogation of large numbers of samples. To determine *PRAME* methylation levels in a large group of normal and tumor samples, we used quantitative bisulfite pyrosequencing [44]. We measured methylation at three CpG sites that showed a clear association with *PRAME* expression in bisulfite clonal sequencing data (Figure 4A; boxed area within Region 3). Figure 5A shows the results of this analysis, which revealed frequent and significant hypomethylation of *PRAME* in EOC as compared to OSE or FTE. Importantly, *PRAME* methylation was inversely correlated with *PRAME* expression (Figure 5B). As an additional test of this association, we obtained an independent set of data from a recent study of HGSC in which *PRAME* methylation was determined using Illumina 450 K bead arrays [5]. The stars in Figure 4A (top) indicate the 11 sites of overlap between bisulfite clonal sequencing data and 450 K data (3 additional sites measured by 450 K are located further upstream). This analysis confirmed an indirect relationship between *PRAME* expression and methylation (Figure 5C). We additionally observed that *PRAME* was significantly hypomethylated in EOC in both early and late stages, in grades 2 and 3 disease, and in tumors with both serous and non-serous histology (Figure 6). We did not observe an association between *PRAME* promoter methylation and OS or DFS (data not shown).

### *PRAME* promoter methylation, *PRAME* mRNA expression, and *LINE-1* methylation

**Figure 7:**
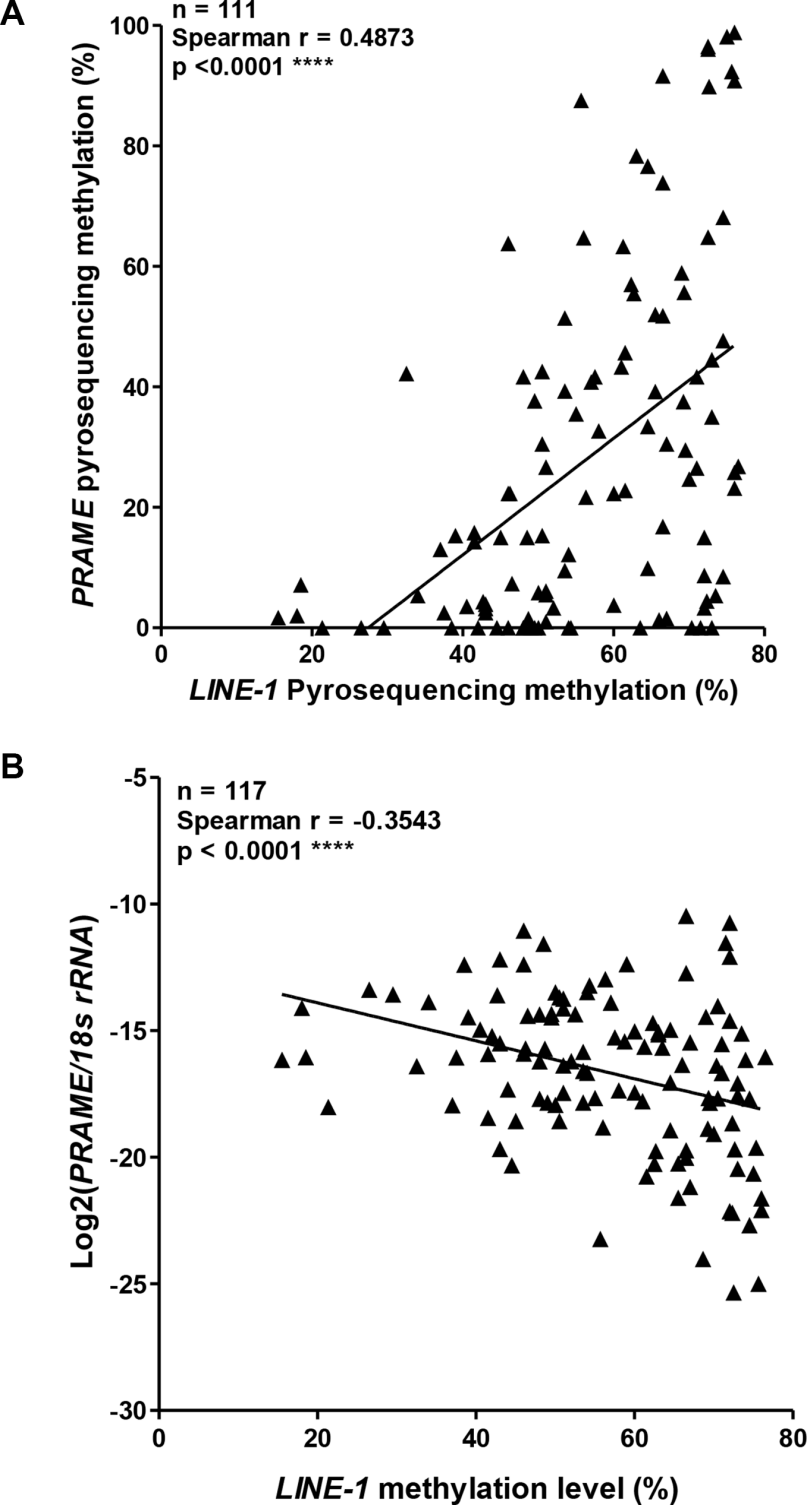
*PRAME* methylation and mRNA expression, and *LINE-1* methylation in EOC (**A**) *PRAME* promoter methylation compared to *LINE-1* methylation in EOC. All methylation data were obtained by pyrosequencing. (**B**) *PRAME* mRNA expression and *LINE-1* methylation in EOC. *PRAME* mRNA expression was determined by RT-qPCR and normalized to *18s rRNA* expression. *LINE-1* methylation was determined by pyrosequencing. In both panels, Spearman test results (two-tailed) are shown.

For several CTA genes, promoter hypomethylation and expression are connected to an epigenetic alteration known as global DNA hypomethylation, a cancer phenotype associated with hypomethylation at repetitive DNA elements [12, 41, 45]. Thus, CTA gene promoters and repetitive elements, both of which are hypermethylated in normal tissues, can become hypomethylated in concert in cancer. To address whether *PRAME* is linked to this phenomenon, we measured *LINE-1* methylation, a surrogate for global methylation status [41], and performed correlation analyses of *LINE-1* methylation with *PRAME* methylation and mRNA expression. Notably, *LINE-1* methylation correlated directly with *PRAME* promoter methylation and indirectly with *PRAME* expression (Figure 7A–7B). Thus, *PRAME* fits within the paradigm previously observed for other X-linked and autosomal CTA genes [41].

### Pharmacological or genetic inhibition of DNMTs induces *PRAME* expression

**Figure 8:**
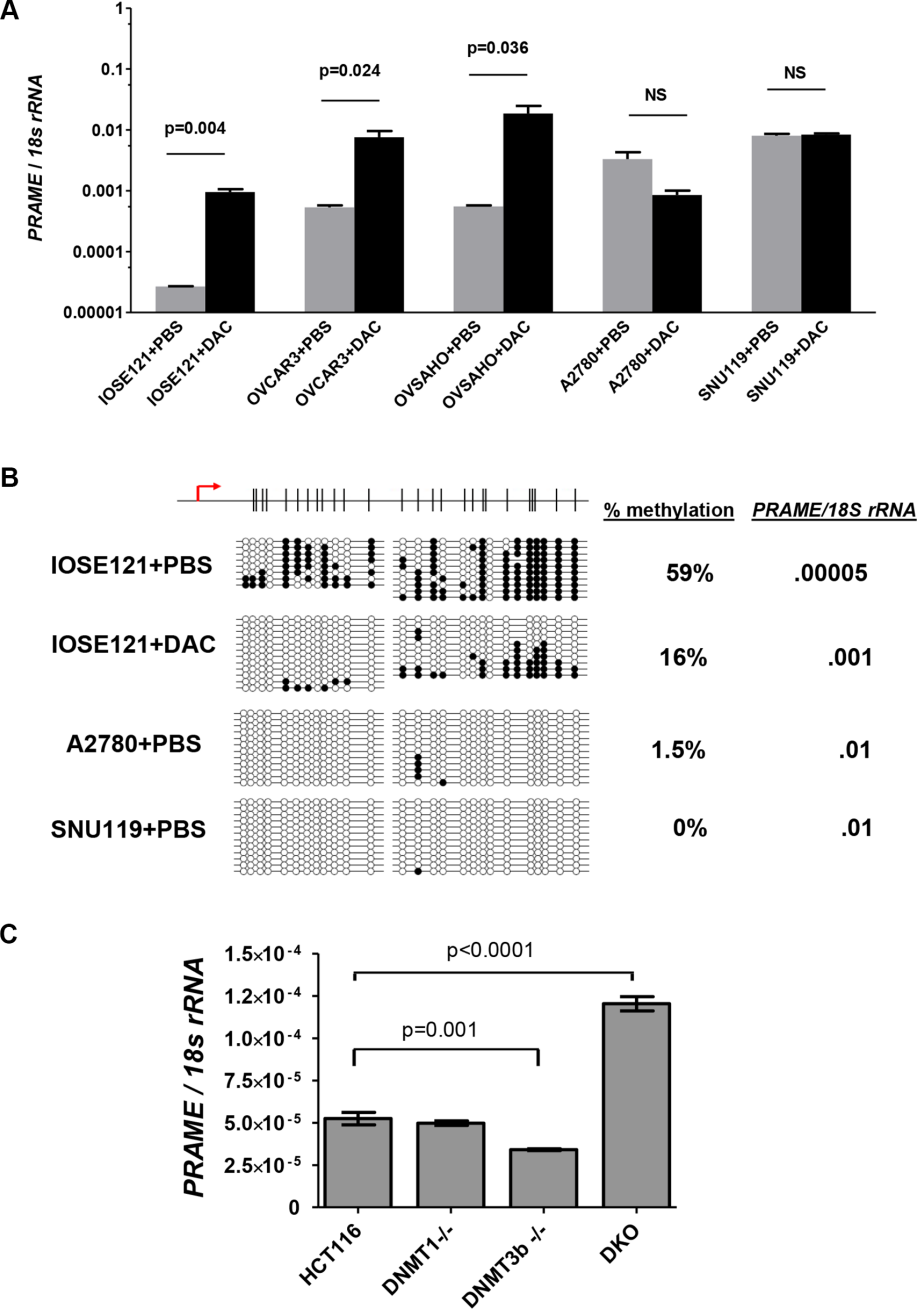
*PRAME* expression in decitabine (DAC)-treated and DNMT knockout cells (**A**–**B**) IOSE121, OVCAR3, OVSAHO, A2780, and SNU119 cell lines were treated with vehicle (PBS) or 1 μM decitabine (DAC) for 5 days as described in *Methods*. A. *PRAME* mRNA expression, determined by RT-qPCR. Data represent mean + SD. Paired two-tailed *t*-test results are shown. (B) Sodium bisulfite clonal sequencing results of the *PRAME* 5′ region in IOSE121 cells after vehicle (PBS) or DAC treatment, and in A2780 and SNU119 cells after PBS treatment. The TSS and CpG map is shown at top. The percent methylation of all sequenced alleles, and the corresponding level of *PRAME* mRNA expression, is indicated at right. (**C**) *PRAME* mRNA expression was measured by RT-qPCR in wild-type HCT116 cells, DNMT1−/−, DNMT3b−/−, and double knockout (DKO) cells. Two-tailed unpaired *t*-test results are shown.

To test whether DNA methylation functionally represses *PRAME* expression, we used decitabine treatment [46]. We treated SV40 large T antigen-immortalized OSE cells (IOSE121) and four EOC cell lines (OVCAR3, OVSAHO, A2780, SNU119). Decitabine treatment significantly induced *PRAME* expression in IOSE121, OVCAR3, and OVSHAO, which showed low baseline *PRAME* expression. In contrast, decitabine did not induce *PRAME* in A2780 or SNU119, which showed high baseline *PRAME* expression (Figure 8A). To confirm that decitabine induced hypomethylation, we conducted bisulfite clonal sequencing of IOSE121 cells and observed hypomethylation, as expected (Figure 8B). In addition, bisulfite sequencing revealed that *PRAME* was hypomethylated at baseline in the two cell lines with high basal *PRAME* expression, A2780 and SNU119 (Figure 8B). To confirm a functional link between DNMTs and *PRAME* expression, we measured *PRAME* in the HCT116 DNMT somatic cell knockout system [47]. Cells with dual knockout of DNMT1 and DNMT3b, but not either enzyme alone, leads to global DNA hypomethylation in these cells [47, 48]. Notably, dual DNMT knockout cells showed elevated *PRAME* expression (Figure 8C).

### PRAME protein expression in EOC

**Figure 9:**
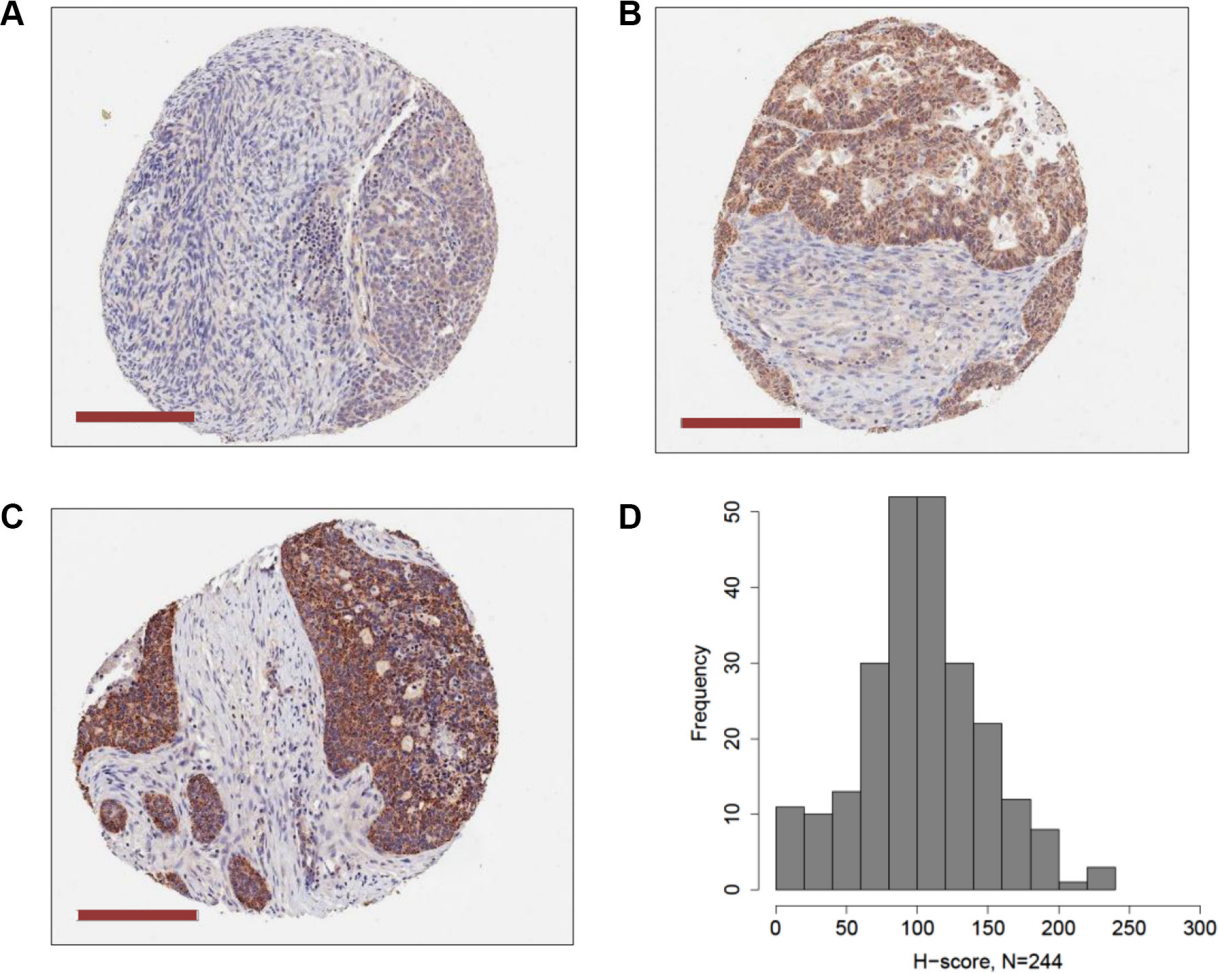
PRAME protein expression in EOC PRAME protein expression was measured by IHC of EOC TMAs as described *Methods*. Examples of (**A**) weak, (**B**) moderate, and (**C**) strong PRAME staining is shown (magnification bars = 200 μM). PRAME was expressed in the cytoplasm of epithelial cells, but not stroma. (**D**) A weighted index score for PRAME cytoplasmic staining (H-score, scale 0–300) for each tumor core was calculated as described in *Methods*. The distribution of H-scores in EOC samples (*N* = 244) is shown. Panels (A–C) are approximately equivalent to H-scores of 90, 185, and 290, respectively.

We used IHC of EOC tissue microarrays (TMA) to measure PRAME protein expression (*N* = 244) and to test its association with *PRAME* mRNA expression, *PRAME* promoter methylation, and clinicopathology. IHC of normal tissues indicated that PRAME was strongly expressed in testis but weakly expressed in most normal tissues, as reported previously [6] (data not shown). EOC showed widespread but variable levels of PRAME expression, and the staining was mostly cytoplasmic. Examples of weak, moderate, and strong PRAME staining in EOC are shown in Figure 9A–9C. Within tumors cores, PRAME was expressed in the epithelial cells but not stroma. We calculated a weighted index of PRAME cytoplasmic staining (H-score, scale 0–300), for each tumor, as described in *Methods*. The distribution of H-scores clustered around 100, indicating that most EOC express PRAME, but do so at relatively low levels (Figure 9D). PRAME H-score directly correlated with *PRAME* mRNA expression and indirectly with *PRAME* methylation, but the latter was not statistically significant (Table 1). PRAME protein expression was not associated with EOC clinicopathology, including survival (Table 1). Sub-group analysis using only HGSC data also indicated no association with survival (Table 1).

**Table 1:** PRAME protein expression vs. molecular and clinical parameters in EOC

	*PRAME mRNA/18S rRNA* (RT-qPCR)	*PRAME* methylation (pyro)		
PRAME protein (IHC H-Score^1^)	0.287 (Spearman) *p* = 0.033 *N* = 55	−0.062 (Spearman) *p* = 0.686 *N* = 45		
			
**ALL EOC (*N* = 244)**		**Low H-Score^2^*N* = 122**	**High H-Score *N* = 122**	***P*-value**
**Age**		62.5	64.0	0.390
**Primary**	Multiple Ovary Primary Peritoneal Fallopian Tube	1 89 27 1	4 99 15 4	0.118
**Stage**	IA, IB, IC, IIC, IIIB IIIC, IV	25 94	25 96	1.000
**Grade**	1, 2 3, 4	21 95	27 100	0.439
**Histology**	Serous Clear Cell Endometrioid Mucinous Other	81 8 7 9 17	90 4 6 3 19	0.288
**Debulking**	Optimal Sub	48 8	50 16	0.250
**Response to Primary Treatment**	Complete Otherwise	62 41	62 31	0.429
**Platinum Sensitivity**	Sensitive Resistant/Refractory	52 30	51 31	1.000
**PFS** **OS**	Median Months (95%CI)	18.5 (13.7–24.3) 39.6 (32.2–52.7)	15.3 (12.8–17.8) 49.7 (39.8–67.0)	0.227 0.097
				
**HGSC (*N* = 160)**		**Low H-Score** ***N* = 76**	**High H-Score** ***N* = 84**	***P*-value**
**PFS** **OS**	Median Months (95%CI)	18.1 (13.7–24.3) 51.1 (41.8–77.1)	15.3 (12.8–17.9) 41.7 (33.6–53.2)	0.408 0.227

1 H-score (range = 0 to 300) is the extent of cytoplasmic immune staining, and = 3× % strongly staining cytoplasm + 2× % moderately staining cytoplasm + % weakly staining cytoplasm.

2 Low and high H-score groups were split using the median H-score value, 88.3.

### *PRAME* expression and RA pathway alterations in EOC

**Figure 10:**
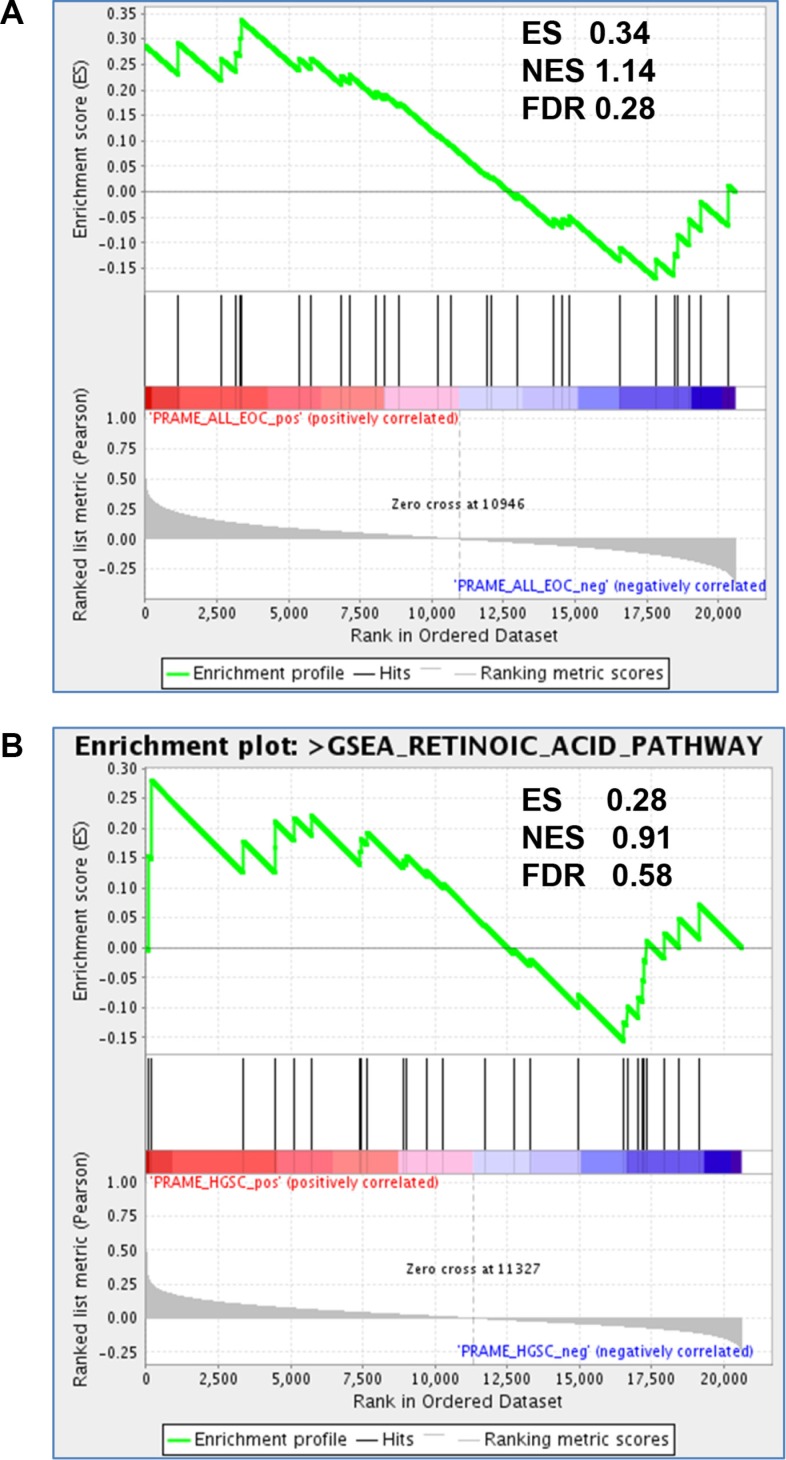
*PRAME* expression and RA pathway signatures in EOC GSEA analysis of enrichment of RA pathway genes (*n* = 30) as a function of *PRAME* expression in (**A**) EOC (*N* = 285) and (**B**) HGSC (*N* = 218), using Affymetrix expression data from [50]. Enrichment score (ES), normalized enrichment score (NES), and false discovery rate *q* values (FDR) are shown. Significant enrichment of RA pathway genes with *PRAME* expression was not observed.

PRAME is a reported inhibitor of RA signaling [8, 24, 25]. In addition, the RA pathway is defective in many cancers, including EOC [49]. To test whether *PRAME* expression is associated with altered RA signaling in EOC and HGSC, we used Gene Set Enrichment Analysis (GSEA) to test the relationship between *PRAME* mRNA expression and RA-mediated gene expression in a large set of published EOC (*N* = 285) and HGSC (*N* = 218) expression data [50]. As shown in Figure 10, this analysis did not strongly support an association between *PRAME* expression and altered RA signaling in EOC or HGSC.

## DISCUSSION

We show here that the CTA *PRAME* is frequently expressed in EOC and HGSC at the mRNA and protein levels, and demonstrate that promoter DNA hypomethylation is a key factor leading to its expression. Correlation analyses linked *PRAME* expression and promoter hypomethylation in primary tumors, and functional studies demonstrated that decitabine treatment and genetic disruption of DNMTs induced PRAME. *PRAME* expression and promoter hypomethylation were associated with *LINE-1* hypomethylation, suggesting that the global hypomethylation phenotype often observed in EOC is connected to *PRAME* regulation, analogous to other CTA genes [13, 41, 42, 51, 52].

In addition to DNA methylation, an epigenetic mechanism, we investigated whether CNA, a genetic mechanism, contributes to *PRAME* expression in HGSC. Although *PRAME* expression directly correlated with copy number, a minority of HGSC cases showed copy number gain or amplifications (16%), and copy number loss was more a frequent alteration (64%), suggesting that CNA makes a minor contributor to increased *PRAME* expression in HGSC. In contrast, *PRAME* promoter hypomethylation was a highly prevalent phenotype (~85% of EOC). We recently reported that a different CTA gene, *CT45*, is also frequently hypomethylated in EOC, but rarely shows copy number gains [42]. These findings support the contention that epigenetic mechanisms are the major ones underlying increased CTA gene expression in EOC. Whether other epigenetic regulatory mechanisms described for CTAs, e.g. histone modifications and nucleosome occupancy [51, 53, 54], also regulate *PRAME*, is a topic of future interest.

*PRAME* expression and promoter hypomethylation were apparent in tumors of both early and late stage and of different grades, using samples comprised of diverse EOC sub-types. To test whether *PRAME* expression correlated with patient survival we used TCGA data, which is comprised only of HGSC. Somewhat surprisingly, this analysis indicated that *PRAME* expression correlated with improved OS and DFS. However, one caveat is that only for *PRAME* microarray data did this effect reach statistical significance, while RNAseq did not. A possible explanation for the improved survival seen with *PRAME* mRNA expression is that PRAME may be immunogenic in EOC patients, and this immunogenicity could drive reduced disease burden [6, 10, 21]. This hypothesis should be tested using studies of PRAME specific antibodies and T-cell responses, and by determining their relationship to patient survival.

Consistent with RT-qPCR analysis of *PRAME*, IHC analysis indicated that the PRAME protein is frequently expressed in EOC. However, PRAME protein expression levels were low to moderate in most tumors. This observation suggests that strategies to increase PRAME expression, for example by treatment with decitabine, could be useful in the context of PRAME-directed immunotherapy [12, 23]. Similar to *PRAME* mRNA expression, we did not observe a significant association between PRAME protein expression and EOC clinicopathological measures, including stage and grade. While the correlation between PRAME mRNA and protein was statistically significant, it was relatively modest (Spearman *r* = 0.287), suggesting that post-transcriptional mechanisms may be important regulators of PRAME protein levels.

Although PRAME was reported to be a negative regulator of RA signaling, our GSEA analysis in EOC or HGSC did not provide support this hypothesis. However, we were restricted to analyzing *PRAME* mRNA expression, which is not fully reflective of PRAME protein expression, as discussed above. Functional studies will be required to clarify whether PRAME plays a role in RA pathway regulation in EOC.

In summary, our study reveals that PRAME is frequently expressed in EOC and HGSC, and establishes it as a potential candidate for immunotherapy in ovarian cancer. The observed regulation of PRAME expression by DNA methylation, coupled with low protein expression in most tumors, raises the possibility that epigenetic modulators such as decitabine could be used to augment PRAME immunotherapy.

## MATERIALS AND METHODS

### Human tissues

We obtained NO, OSE, FTE, and EOC tissues using IRB-approved protocols at RPCI as described previously [41, 55]. We prepared tissue extracts as described previously [13]. EOC tissues were estimated by pathology to contain > 80% neoplastic cells.

### Human cell lines

We obtained and cultured SV40 Large-T antigen-immortalized normal human OSE cells, IOSE121, and EOC cell lines A2780 and OVCAR3 as described previously [13]. We obtained and cultured SNU119 and OVSAHO HGSC cells as described previously [39]. We obtained HCT116 wild-type, HCT116 DNMT1−/−, HCT116 DNMT3b−/−, and HCT116 DNMT1−/−, 3b−/− (DKO) cells from Dr. Bert Vogelstein [47], and cultured as described previously [48].

### DNA and RNA extractions

We isolated genomic DNA (gDNA) using the Puregene Kit A (Qiagen), and purified total RNA using TRIzol^®^ (Invitrogen).

### Decitabine (DAC) treatment

We obtained decitabine from Sigma. We treated cells at ~70% confluence with 1 μM DAC (day 0), passaged cells at day 2, re-treated with 1 μM DAC at day 3, and harvested cells for RNA and gDNA extracts at day 5.

### Reverse transcriptase quantitative PCR (RT-qPCR)

We processed RNA using the DNA-free kit (Ambion), and performed cDNA synthesis using the iScript cDNA Synthesis Kit (BioRad). We measured *PRAME* expression by qPCR using a BioRad CFX Connect system and the Sybr green method. We prepared standard curves using a mixture of cDNA from EOC cell lines, and normalized *PRAME* mRNA expression to *18s rRNA*. Primer sequences are provided in Supplementary Table 1.

### EOC TMA and PRAME IHC

EOC TMA were described previously [42, 56]. TMA sections were cut to 4 μm, placed on charged slides, and dried at 60°C for one hour. Slides were cooled to room temperature, de-paraffinized in three changes of xylene, and rehydrated using graded alcohols. For antigen retrieval, slides were heated in a steamer for 40 minutes in EDTA buffer, pH = 8, (Lab Vision), followed by a 20-minute cool down. Endogenous peroxidase was quenched with aqueous 0.3% H_2_O_2_ for 10 minutes and washed with PBS/T. Slides were loaded on a DAKO autostainer (Dako) and serum-free protein block was applied for 5 minutes, blown off, and then PRAME primary antibody (Sigma, catalog no. HPA045153) was applied at 1.3 μg/ml for one hour. A matched isotype antibody was applied to a replicate slide, in place of primary antibody, as a negative control. The EnVision+ horseradish peroxidase system (Dako) and DAB chromogen were used for visualization. Slides were counterstained with hematoxylin, dehydrated, cleared and cover slipped.

### Aperio slide scanning and IHC image analysis

TMA slides were digitally scanned using Aperio Scanscope (Aperio Technologies) with 20x bright-field microscopy. These images are then accessible using Spectrum (Aperio Technologies). Once slides are scanned, Aperio ImageScope version 11.2.0.780 was used to view images for image analysis. Images were examined for quality and were amended as necessary. An annotation layer was created for each TMA core. Tumor regions were identified and annotated to appropriately represent the heterogeneity of staining for image analysis. The Aperio platform was used to develop quantitative image analysis algorithm macros for quantification of IHC. The cytoplasmic algorithm was modified to detect and quantify the positive DAB staining cells. The intensity for the positive stain was divided into four score values, 0-none, 1+- weak, 2+-moderate, and 3+-strong. For each cytoplasm threshold, the percentage of cells having staining in the cytoplasm was provided, and the overall percentage of positive cells and average intensity score were calculated for each layer. In addition, the algorithm provided an H-Score. The H-score is a cytoplasmic intensity score derived from the average intensity of the staining of the cytoplasm, according to the threshold intervals set in the algorithm macro. H-score = 1*(%1+) + 2*(%2+) + 3*(%3+), with the score between 0 and 300, where 300 represents 100% of cells being 3+. PRAME H-scores were averaged over 1–6 slides for each patient (*N* = 244).

### DNA methylation analyses

We used sodium bisulfite clonal sequencing and sodium bisulfite pyrosequencing to determine the DNA methylation status of the *PRAME* 5′ region [44, 57]. We performed bisulfite conversions using the EZ DNA Methylation Kit (Zymo Research) and designed bisulfite sequencing primers using MethPrimer. We determined *LINE-1* repetitive element DNA methylation using bisulfite pyrosequencing as described previously [40]. Primer sequences are reported in Supplementary Table 1.

### *PRAME* mRNA expression and HGSC survival

We retrieved TCGA HGSC *PRAME* mRNA expression datasets [Affymetrix U133 microarray (*N* = 576), Agilent microarray (*N* = 561), and RNAseq (N = 307)] and patient survival data using cBioPortal [36, 37]. We performed survival analyses using Graphpad Prism.

### PRAME protein expression vs. EOC clinicopathology

Overall Survival (OS) was defined as the time between the date of diagnosis and death. Patients who were alive at the time of analysis were censored at the date of last follow up. Progression-free survival (PFS) was defined as the time between the date of surgery and disease recurrence. Patients who were alive and disease-free were censored at the date of last follow-up and median survival times were estimated from the Kaplan-Meier curve. Association between PRAME H-score and clinical parameters was tested with a combination of chi-square and *t*-tests as appropriate; all tests were two-sided.

### *PRAME* copy number and mRNA expression in TCGA HGSC data

We retrieved TCGA copy number data for HGSC and other tumor types from cBioPortal [36, 37], and obtained GISTIC putative copy-number alterations using Onco Query Language. We obtained *PRAME* mRNA expression data (RNA-seq, *N* = 300; Affymetrix U133 microarray, *N* = 556) from cBioPortal.

### *PRAME* methylation and mRNA expression from International Cancer Genome Consortium (ICGC) primary tumor HGSC data

The Ovarian Cancer - Australia (OV-AU) DNA methylation (Illumina 450 K methylation) and gene expression (Illumina RNAseq) datasets, both version 2015-07-15, were downloaded from UCSC Xena (https://genome-cancer.soe.ucsc.edu/proj/site/xena/heatmap/). Statistical analysis was performed with Graphpad prism. This data set has been described [5].

### GSEA analysis for RA pathway signatures

We performed GSEA http://www.broadinstitute.org/gsea/index.jsp) to test for enrichment of RA pathway network genes (http://software.broadinstitute.org/gsea/msigdb/geneset_page.jsp?geneSetName=PID_RETINOIC_ACID_PATHWAY, *N* = 30 genes) with *PRAME* expression in EOC (*N* = 285) or HGSC (*N* = 218). We used a continuous phenotype model, based on *PRAME* expression data obtained from Affymetrix U133 Plus 2 arrays (GEO accession GSE9899) [50].

### Statistical analyses of molecular data

We used Graphpad Prism for standard statistical analyses to compare molecular parameters between sample groups. The statistical test used and *p*-values are provided in the figures and figure legends.

## Supplementary Materials




