# Supplementary material for: PRAME expression and promoter hypomethylation in epithelial ovarian cancer
Source: Oncotarget. 2016 Jun 13;7(29):45352–69. doi: 10.18632/oncotarget.9977 (PMC5216727; doi:10.18632/oncotarget.9977)
Supplement: Supplementary file 1 [file oncotarget-07-45352-s001.pdf]

PRAME expression and promoter hypomethylation in epithelial ovarian cancer

Supplementary Materials

Supplementary Table S1: Primer sequences. See Supplementary_Table_S1
